# Super-Resolution Arterial Spin Labeling Using Slice-Dithered Enhanced Resolution and Simultaneous Multi-Slice Acquisition

**DOI:** 10.3389/fnins.2021.737525

**Published:** 2021-10-29

**Authors:** Qinyang Shou, Xingfeng Shao, Danny J. J. Wang

**Affiliations:** Laboratory of FMRI Technology, USC Mark and Mary Stevens Neuroimaging and Informatics Institute, Keck School of Medicine, University of Southern California, Los Angeles, CA, United States

**Keywords:** super-resolution (SR), arterial spin label (ASL) MRI, perfusion, simultaneous multi slice, slice dithered enhanced resolution (SLIDER)

## Abstract

**Purpose:** To achieve high spatial resolution (isotropic-2 mm) perfusion imaging using 2D simultaneous multi-slice (SMS) pseudo-continuous arterial spin labeling (pCASL) and slice dithered enhanced resolution (SLIDER) technique for super-resolution reconstruction.

**Methods:** The SLIDER-SMS pCASL with a multiband factor of 4 was implemented at 3T with three numbers of slice shift (2/3/4) for the slice thickness of 4/6/8 mm, respectively. Super-resolution reconstruction was performed with singular value decomposition and different levels of Tikhonov regularizations. Temporal and spatial signal-to-noise ratio (SNR) as well as spatial blurring effects of super-resolution ASL images were measured in five healthy subjects and compared with those of reference high-resolution ASL images.

**Results:** Compared to conventional 2D SMS ASL, super-resolution ASL images with isotropic-2-mm resolution yielded 42, 61, and 88% higher spatial SNR, and 18, 55, and 105% higher temporal SNR with slice shift number of 2/3/4, respectively. Spatial blurring effect increased for SLIDER reconstruction from two to four slice shifts.

**Conclusion:** The proposed SLIDER-SMS pCASL technique can achieve whole-brain high-resolution perfusion images with ∼15-min scan time and improved SNR compared to standard 2D SMS pCASL. Caution needs to be exercised on quantifying and controlling blurring effects of SLIDER reconstruction.

## Introduction

Arterial spin labeling (ASL) is a perfusion imaging technique that can quantitatively measure cerebral blood flow (CBF) without using an exogenous contrast agent. Due to the relatively low signal-to-noise ratio (SNR), existing ASL methods generally have a coarse resolution of 3–4 mm resulting in partial volume effects when analyzing perfusion of small brain structures and cerebral cortex with a thickness of a few mm. Segmented 3D acquisitions and pseudo-continuous ASL (pCASL) are recommended for ASL imaging to achieve a sufficient SNR(Alsop et al., 2015). However, there remain challenges for achieving a high spatial resolution with 3D ASL including spatial blurring along the slice direction due to the modulation of k-space signals by the transverse (T2) relaxation and susceptibility to (intersegment) head motion.

Simultaneous multi-slice (SMS) is a fast imaging technology that simultaneously excites multiple slices and resolves each slice with parallel imaging techniques (Feinberg et al., 2010; Setsompop et al., 2012). When applied to ASL imaging, SMS can reduce the effect of signal decay due to longitudinal (T1) relaxation and improve spatial coverage and/or resolution compared to standard 2D ASL (Feinberg et al., 2013; Kim et al., 2013; Li et al., 2015; Wang et al., 2015). Compared to 3D imaging, 2D SMS has the potential benefits of reduced spatial blurring and improved robustness to head motion. Furthermore, a constrained slice-dependent background suppression (CSD-BS) scheme has been proposed to suppress the background signal in brain tissue to improve the SNR for 2D SMS pCASL (Shao et al., 2018).

Super-resolution techniques achieve high spatial resolution from one or multiple low-resolution images with reconstruction algorithms, which have been proven effective in MRI (Ben-Eliezer et al., 2010; Setsompop et al., 2018; Vu et al., 2018; Greenspan et al., 2002). The SLIce Dithered Enhanced Resolution (SLIDER) (Setsompop et al., 2015) technique is a super-resolution technique that utilizes sub-voxel spatial shifts in the slice direction to achieve a √*n* fold gain (*n* is the number of spatially shifted thick slices) in SNR efficiency, given the same number of measurements and without considering physiological noise or parallel imaging factors. SLIDER has been successfully applied for SMS diffusion MRI and BOLD fMRI to achieve submillimeter spatial resolutions (Setsompop et al., 2015; Vu et al., 2018). This method may provide a potential technique for high-resolution ASL by increasing both resolution and SNR efficiency while still achieving a wide imaging coverage.

The purpose of this study was to present a super-resolution (iso-2-mm) perfusion imaging technique by integrating SLIDER with 2D SMS pCASL and CSD-BS, herein termed as SLIDER-SMS pCASL. We first presented the theoretical framework and then demonstrated *in vivo* experimental results by comparison with a reference high-resolution 2D SMS pCASL.

## Materials and Methods

### Theoretical Framework

**FIGURE 1:**
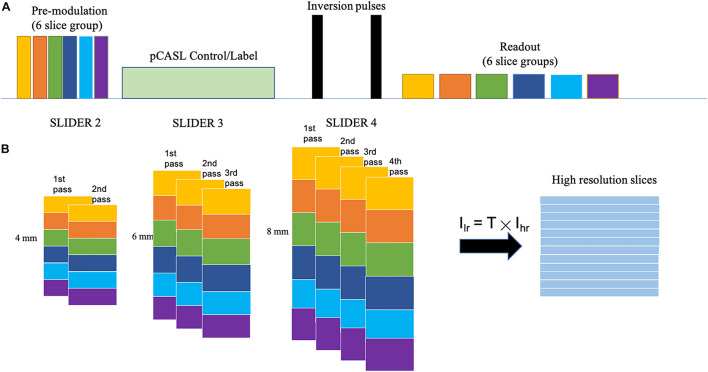
SLIDER acquisition scheme. **(A)** SMS-EPI pCASL acquisition sequence for each set of low-resolution images, with constrained slice dependent background suppression. **(B)** Acquisition scheme for SLIDER2, SLIDER3, and SLIDER4; different numbers of scans with slice shift are needed, 2 for SLIDER2, 3 for SLIDER3, and 4 for SLIDER4. Then, high-resolution images can be reconstructed.

**FIGURE 2:**
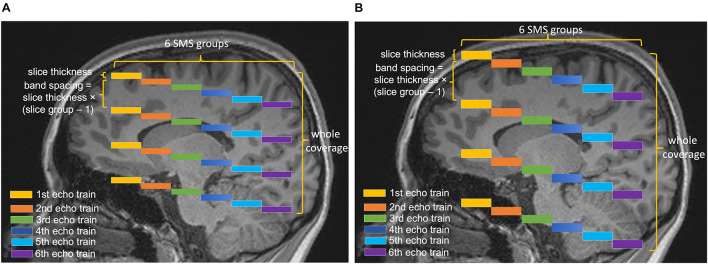
Diagram of the SMS scheme. Six slice groups with a total slice number of 24 are acquired in each TR. The band spacing is adjusted according to the slice thickness to avoid slice cross talk. The center is aligned in different conditions, so that the slice thickness of **(A)** 4 mm and **(B)** 8 mm will have different coverages in the slice direction.

Figure 1 shows the diagram for SLIDER-SMS pCASL which combined SMS imaging, SLIDER super-resolution with the optimized CSD-BS scheme (Shao et al., 2018). In this study, six SMS slice groups with a multiband (MB) factor of 4 were employed to achieve whole-brain coverage. The detailed SMS scheme is shown in Figure 2. In SLIDER, *N* sets of 2D slices are acquired with sub-voxel spatial shifts in the slice direction, and high-resolution thin-slice images are reconstructed by applying a “deblurring” algorithm. Three options for SLIDER acquisition are shown in Figure 1B, namely, SLIDER2, SLIDER3, and SLIDER4 for two, three, and four sets of shifted slices with the thickness of 4, 6, and 8 mm, respectively. In our study, the reference high-resolution SMS pCASL images with 2-mm slice thickness (termed as reference ASL images hereafter) were acquired in two scans, each with a 100% inter-slice gap, to match the image acquisition time of super-resolution scans and to minimize cross talk between thin slices. The CSD-BS method employs slice-dependent pre-modulation MB RF pulses for each group of SMS slices before the pCASL pulse train as well as two inversion pulses during the post-labeling delay (PLD), so that the longitudinal magnetization (M_*z*_) of each group of SMS slices reaches the nulling point just before the readout of each respective slice group.

The theoretical SNR for the super-resolution images (SNR_*SLIDER–SMS*_) reconstructed with this method compared to the SNR of reference ASL images (SNR_*ref*_) with the same number of measurements and without considering physiological noise or parallel imaging factors can be expressed by the following equation:


(1) $$S\,N\,R_{S\,L\,I\,D\,E\,R-S\,M\,S}=\sqrt{N_{s\,e\,t}}\times S\,N\,R_{r\,e\,f}$$

where *N*_*set*_ is the number of shifted image sets, since the potential thin slices have been sampled for *N*_*set*_ times.

However, this equation only applies to non-SMS-accelerated acquisitions. For SLIDER-SMS pCASL, image noise caused by g-factor should be considered and can be expressed as the following equation:


(2) $$v\,a\,r_{S\,L\,I\,D\,E\,R-S\,M\,S}=g_{S\,L\,I\,D\,E\,R-S\,M\,S}^{2}\times v\,a\,r_{t\,h\,e\,r\,m\,a\,l}+v\,a\,r_{p\,h\,y\,s\,i\,o}$$

where *g_SLIDER__–SMS_* is the g-factor for SLIDER-SMS reconstruction (Breuer et al., 2009), *var* represents noise variance, and the subscript represents the respective physiological and thermal noise. When *N* measurements (*N*_*meas*_) are averaged for ASL, the image noise variance can be expressed by


(3) $$v\,a\,r_{S\,L\,I\,D\,E\,R-S\,M\,S,N_{m\,e\,a\,s}}=g_{S\,L\,I\,D\,E\,R}^{2}\times \frac{v\,a\,r_{t\,h\,e\,r\,m\,a\,l}}{N_{m\,e\,a\,s}}+\frac{v\,a\,r_{p\,h\,y\,s\,i\,o}}{N_{m\,e\,a\,s}^{\prime }}$$

where *N′_*meas*_* is between 1 and *N*_*meas*_ depending on the temporal correlation of physiological noise. The labeling duration and PLD of an ASL scan are much longer than the image acquisition time. In order to keep the total scan time constant for SLIDER2/3/4 and reference ASL scans (for fair comparison of SNR efficiency), we used fewer repetitions for SLIDER acquisitions with more shifted slices (*N*_*set*_). For example, in SLIDER2, two scans were acquired with 40 repetitions in each scan, whereas in SLIDER4, four scans were acquired with only 20 repetitions in each scan. Therefore, the SNR efficiency for the proposed SLIDER-SMS pCASL can be expressed by


(4) $$S\,N\,R_{S\,L\,I\,D\,E\,R-S\,M\,S}=\sqrt{N_{s\,e\,t}}\times S\,N\,R_{r\,e\,f,S\,M\,S}\times \sqrt{\frac{\frac{v\,a\,r_{p\,h\,y\,s\,i\,o}}{N_{f\,u\,l\,l}^{\prime }}+g_{r\,e\,f,S\,M\,S}^{2}\times \frac{v\,a\,r_{t\,h\,e\,r\,m\,a\,l}}{N_{f\,u\,l\,l}}}{\frac{v\,a\,r_{p\,h\,y\,s\,i\,o}}{N_{m\,e\,a\,s}^{\prime }}+g_{S\,L\,I\,D\,E\,R-S\,M\,S}^{2}\times \frac{v\,a\,r_{t\,h\,e\,r\,m\,a\,l}}{N_{m\,e\,a\,s}}}}$$

where *N*_*full*_ is the number of measurements of the reference ASL scan, *N′_*full*_* is between 1 and *N*_*full*_ depending on the temporal correlation of physiological noise, and *g_ref_,_SMS_* is the g-factor for the SMS acquisition of the reference ASL images. Because the coil g-factor generally decreases with larger spacing between slice groups for SMS reconstruction and physiological noise cannot be suppressed as effective as thermal noise by averaging, Eq. (4) predicts that the SNR efficiency of SLIDER ASL will increase with thicker acquired slices and a higher number of shifted slices (*N*_*set*_). This hypothesis will be tested by *in vivo* experiment below.

The reconstruction of SLIDER acquisition can be viewed as solving an inverse problem of the following equation:


(5) $$A\times I_{h\,r}=I_{l\,r}$$

where *I*_*lr*_ is the combined low-resolution images acquired, *A* is the forward model constructed according to the acquisition, and *I*_*hr*_ is the high-resolution images to be reconstructed. In this study, a Toeplitz matrix was used as the forward model, which assumes a perfect slice profile, and singular value decomposition with Tikhonov regularization was used to calculate the pseudo-inversion of the *A* matrix (Setsompop et al., 2018).


(6) $$I_{h\,r}={\left(A^{T}\,A+\lambda \,I\right)}^{-1}\,A^{T}\,I_{l\,r}=A_{i\,n\,v\,\_\,t\,i\,k}\,I_{l\,r}$$

The regularization parameter λ was chosen between 0.1, 0.3, and 0.5 to serve as different regularization levels. A larger regularization parameter will cause less noise amplification but more spatial blurring in the reconstructed images, which will be evaluated below.

### MRI Experiments

Five healthy subjects (age = 24.6 ± 1.7 years, four males) were scanned on a Siemens 3T Prisma scanner using a 32-channel head coil, after they provided written consent according to the protocol approved by the institutional review board of the University of Southern California. The imaging parameters for SLIDER-SMS pCASL included the following: in-plane resolution of 2 mm × 2 mm, field of view (FOV) of 192 mm, matrix size of 96 × 96, TR = 5,000 ms, TE = 20 ms. The MB factor was 4, and the number of slice groups was 6 with 24 total slices acquired for each TR. The acquisition of each SMS slice group took 45 ms, i.e., total 270 ms for six slice groups. Blipped CAIPI was applied to avoid voxel tilting (Setsompop et al., 2012). The labeling duration for pCASL was 1,500 ms, and the PLD was 1,800 ms. The labeling plane was placed 90 mm below the center of the most superior slice group and was adjusted in acquisitions of the shifted slices so that the labeling plane was at the same location across all the conditions. The optimized parameters for CSD-BS including the timing of the two inversion pulses were calculated according to (Shao et al., 2018). Two/three/four sets of low-resolution images with slice thickness of 4, 6, and 8 mm were acquired for SLIDER2/3/4 scans, respectively. The spacing between bands was adjusted according to the slice thickness (20, 30, and 40 mm for slice thickness of 4, 6, and 8 mm, respectively) to avoid inter-slice cross talk. A detailed SMS scheme is shown in Figure 2. A single-band reference scan was acquired before each of the SMS pCASL scans for SMS image reconstruction. For SLIDER2, each of the two SMS pCASL scans had 40 measurements with a duration of 6 min 45 s. For SLIDER3, each of the scan had 26 measurements and the total scan time was 4 min 35 s × 3 = 13 min 45 s. For SLIDER4, each of the scan had 20 measurements and the total scan time was 3 min 20 s × 4 = 13 min 20 s. In this way, the total scan time for each of the SLIDER methods was about 14 min. Reference ASL images were acquired by two interleaved high-resolution SMS pCASL scans (2 mm × 2 mm × 2 mm) with a 100% slice gap in each scan and, with the same background suppression parameters, were acquired as the benchmark. M0 images were acquired before each ASL scan with the same resolution. A high-resolution (iso-0.8 mm) structural MRI was acquired using a T1-weighted MPRAGE scan in each subject.

The raw k-space data of the SMS scans were processed offline using custom Matlab programs. First, raw data were corrected for N/2 phase shift caused by EPI acquisition. Second, the unaliased MB images were reconstructed using slice-GRAPPA (Setsompop et al., 2012) with a kernel size of 5 × 5 derived from the single-band references. Then, each scan was corrected for rigid head motion using SPM12^1^ and combined into a full data matrix. SLIDER reconstruction was applied to achieve 2-mm isotropic M0 and perfusion images according to Eq. 6 with three different λs (0.1, 0.3, and 0.5). The numbers of 2-mm slices reconstructed were 48, 48, 72, and 96 for reference ASL and SLIDER2/3/4 scans, respectively. A principal component analysis (PCA)-based denoising algorithm (Shao et al., 2017) was further applied for both super-resolution and reference ASL scans. In our experiment, PCA reduced the overall noise level in all conditions but did not impact the comparison of super-resolution reconstructions. The spatial SNR (sSNR) and temporal SNR (tSNR) values with and without PCA denoising are listed in Supplementary Table 1. In addition, the g-factor maps for each SMS reconstruction of the SLIDER condition were calculated using the definition in Breuer et al. (2009) with the slice-GRAPPA kernel and the measured noise-covariance matrix.

### Data Analysis

Both the reference ASL images and the reconstructed super-resolution images were evaluated by SNR in the gray matter (GM) region of interest (ROI). The GM probability map was obtained by segmentation of the MPRAGE structural images using SPM12, which was co-registered to the reference ASL M0 images. A threshold of 0.9 was applied on the probability map to obtain the GM mask. We used the definition in Feinberg et al. (2013) to calculate SNR. Even- and odd-numbered perfusion images were averaged, and then added and subtracted to generate the sum and difference images. The sSNR was calculated as the mean of the sum image divided by 1/√2 of the standard deviation (SD) of the difference image within the GM ROI. This definition of SNR calculation can reduce the effect of inhomogeneous noise distribution. tSNR was calculated by the mean signal divided by the SD across time frames within the GM ROI. To evaluate the spatial blurring effect of SLIDER reconstructions, Pearson correlation coefficients of the reconstructed image volumes and the volumes shifted one slice (2 mm) down were calculated by collapsing all voxels into a vector. Furthermore, sSNR and tSNR measurements were repeated with intentionally applied spatial smoothing along slice direction on SLIDER2, 3 and reference ASL images to match the spatial blurring of SLIDER4. A nonparametric Kruskal–Wallis test with *post hoc* Wilcoxon signed-rank test was performed to compare SNR measurements between SLIDER and reference ASL scans, while two-way ANOVA and *post hoc* Wilcoxon signed-rank test were performed to compare slice blurring effects.

Pixel-wise correlations were performed between the reconstructed SLIDER images and reference ASL image within the brain mask. Difference images between the SLIDER and reference ASL images were obtained along with histograms of pixel values within the brain mask for each SLIDER condition. One sample *t*-test and Kolmogorov–Smirnov test were performed to test whether the difference images had zero mean and normal distribution, respectively.

Cerebral blood flow maps were calculated for each SLIDER and reference ASL images, respectively. Slice-dependent PLD correction was performed before quantification. Seven small brain regions (caudate, putamen, etc.) were segmented from the T1-weighted image by Freesurfer v7.1.1 and then co-registered to the M0 image of each condition. The mean CBF values were extracted from these ROIs and were compared across experimental conditions using two-way repeated measures ANOVA and *post hoc* Wilcoxon signed-rank test.

## Results

### Slice Dithered Enhanced Resolution-Simultaneous Multi-Slice Pseudo-Continuous Arterial Spin Labeling Perfusion Images

**FIGURE 3:**
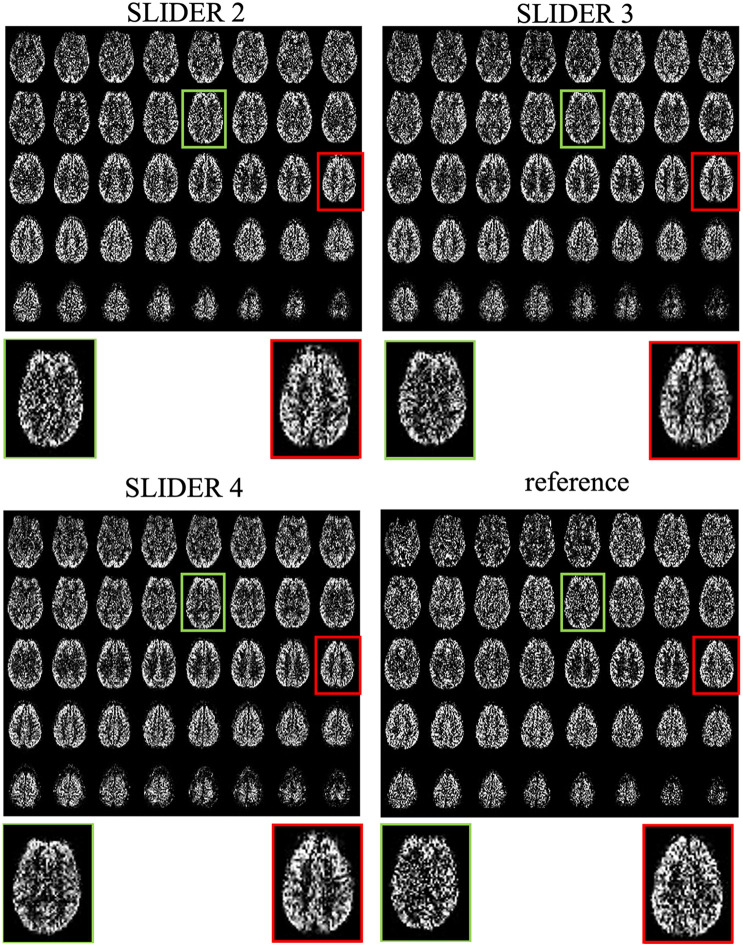
SLIDER super-resolution reconstructed ASL images and acquired reference ASL images in the axial direction. The reconstructed images follow the reference images well, with an improved SNR. The red and green boxes and zoomed images show two slices with SNR improvement.

Figure 3 shows the center 40 axial slices of the reconstructed super-resolution perfusion images with SLIDER2/3/4 and reference ASL images of a representative subject, respectively. All SLIDER reconstructions shown in Figure 3 used a regularization parameter of λ = 0.1. The reconstructed perfusion images for SLIDER2/3/4 scans show good quality without visible artifacts and with clear contrast between gray and white matter as the reference ASL perfusion images. Scatter plots of pixel-wise correlations between the reconstructed SLIDER and reference ASL images are shown in Supplementary Figure 1. There were good correlations with reference ASL images in all three SLIDER conditions (0.73 for SLIDER2, 0.76 for SLIDER3, and 0.73 for SLIDER4, *p* < 0.0001). Supplementary Figure 2 shows the difference images and associated histograms between the SLIDER and reference ASL images. The difference images had zero mean and followed normal distribution (*p* > 0.05 for all three conditions).

**FIGURE 4:**
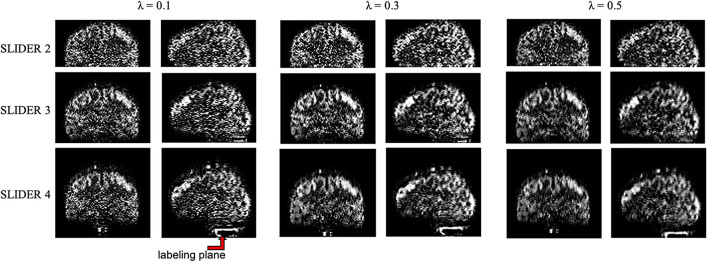
SLIDER super-resolution reconstructed image in the sagittal and coronal direction. SLIDER2 has a brain coverage of 96 mm, SLIDER3 has a brain coverage of 144 mm, and SLIDER4 has a brain coverage of 192 mm. SLIDER4 can reach the labeling plane, as marked by the red arrow. Larger regularization parameters will lead to more spatial blurring.

The zoomed slices show that the SNR of SLIDER reconstructions is higher than the reference ASL images. Figure 4 shows the reconstructed super-resolution perfusion images in sagittal and coronal views with different regularization parameters, respectively. Using our experimental paradigm, the three SLIDER methods had different coverages in the slice direction with the largest coverage in SLIDER4 (96 slices), and narrower coverages in SLIDER3 (72 slices) and SLIDER2 (48 slices). SLIDER4 can even cover the labeling plane, as shown by the red arrow. Figure 4 also shows that as the regularization parameter λ increases, the SNR of the perfusion image increases, but there is more spatial blurring. The full slices of SLIDER2/3/4 ASL images are shown in Supplementary Figure 3.

### Signal-to-Noise Ratio Comparison Between Super-Resolution and Reference Arterial Spin Labeling

**FIGURE 5:**
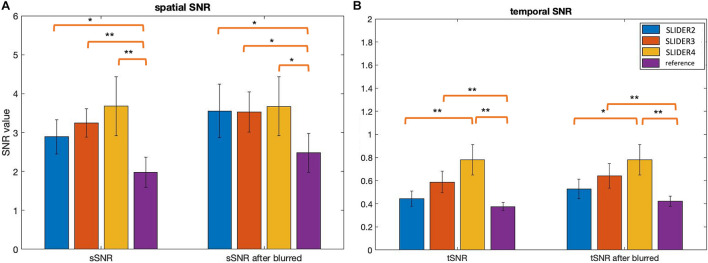
Spatial and temporal SNR before and after controlling slice blurring. **(A)** Spatial SNR (sSNR) and **(B)** temporal SNR (tSNR) of SLIDER2/3/4 and reference scans before (left) and after applying a Gaussian kernel to control slice blurring (right). *p < 0.05; **p < 0.01. Error bars stand for standard deviation.

**FIGURE 6:**
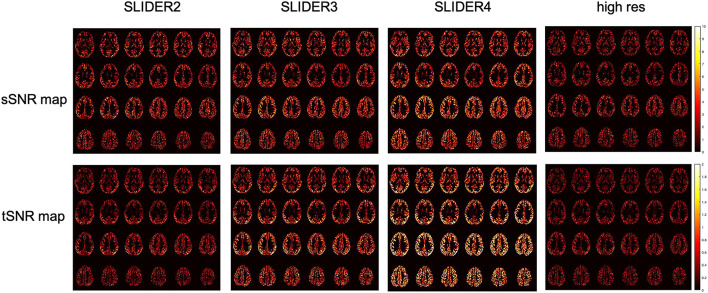
Spatial SNR (sSNR) map and temporal SNR (tSNR) map of a representative subject in different SLIDER conditions and reference ASL scan.

Figure 5 shows the spatial and temporal SNR values of the reconstructed images for all five subjects (λ = 0.1) (the details of each subject are included in Table 1). Figure 6 shows the sSNR and tSNR map of a typical subject. The Kruskal–Wallis test showed significant differences in spatial and temporal SNR across the reconstructed super-resolution images and the reference ASL images (*p* < 0.01 for sSNR and *p* < 0.005 for tSNR). SLIDER4, 3, and 2 improved sSNR by 88, 61, and 42%, respectively, compared to the reference ASL images. TSNR was increased by 105, 55, and 18% using SLIDER4, 3, and 2, respectively, compared to reference ASL images. The trend of such SNR improvement is stable across the five subjects. *Post hoc* Wilcoxon signed-rank test showed that SLIDER can significantly improve sSNR (*p* < 0.05) and tSNR (*p* < 0.01).

**TABLE 1:** Spatial and temporal SNR of the super-resolution reconstructed images and reference images for all five subjects.

	**sSNR**	**sSNR (after blurred)**	**tSNR**	**tSNR (after blurred)**
Sub1 (SLIDER2)	3.06	3.89	0.49	0.59
Sub1 (SLIDER3)	3.19	3.58	0.62	0.68
Sub1 (SLIDER4)	3.68	3.68	0.81	0.81
Sub1 (reference)	2.13	2.72	0.39	0.44
Sub2 (SLIDER2)	3.17	4.01	0.51	0.60
Sub2 (SLIDER3)	3.36	3.77	0.69	0.75
Sub2 (SLIDER4)	3.45	3.45	0.82	0.82
Sub2 (reference)	2.06	2.62	0.40	0.45
Sub3 (SLIDER2)	2.18	2.82	0.37	0.43
Sub3 (SLIDER3)	2.76	3.11	0.48	0.52
Sub3 (SLIDER4)	2.74	2.74	0.59	0.59
Sub3 (reference)	1.35	1.71	0.32	0.35
Sub4 (SLIDER2)	3.28	4.25	0.48	0.58
Sub4 (SLIDER3)	3.77	4.24	0.65	0.72
Sub4 (SLIDER4)	4.84	4.84	0.95	0.95
Sub4 (reference)	2.4	3.04	0.40	0.46
Sub5 (SLIDER2)	2.17	2.81	0.38	0.44
Sub5 (SLIDER3)	2.63	2.96	0.50	0.54
Sub5 (SLIDER4)	3.66	3.66	0.73	0.73
Sub5 (reference)	1.83	2.30	0.37	0.42
Mean ± SD (SLIDER2)	2.77 ± 0.55	3.56 ± 0.69	0.45 ± 0.07	0.53 ± 0.09
Mean ± SD (SLIDER3)	3.14 ± 0.46	3.53 ± 0.51	0.59 ± 0.10	0.64 ± 0.11
Mean ± SD (SLIDER4)	3.67 ± 0.76	3.67 ± 0.76	0.78 ± 0.13	0.78 ± 0.13
Mean ± SD (reference)	1.95 ± 0.40	2.48 ± 0.50	0.38 ± 0.03	0.42 ± 0.05

*“After blurred” means additional Gaussian smoothing in the slice direction has been applied to the image to match the blurring level of SLIDER4.*

The g-factor maps in the sagittal plane for the three SMS acquisitions are shown in Supplementary Figure 4. The mean g-factor value in the brain across five subjects for SLIDER2 is 1.28 ± 0.06, that for SLIDER3 is 1.38 ± 0.07, and that for SLIDER4 is 1.34 ± 0.06.

**FIGURE 7:**
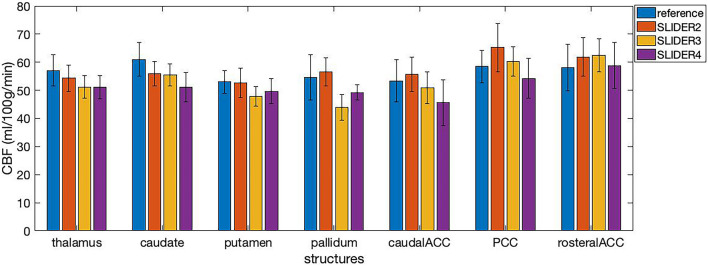
Cerebral blood flow values in seven small brain structures acquired under different SLIDER and reference conditions. ACC, anterior cingulate cortex; PCC, posterior cingulate cortex. Error bars stand for stand deviation.

The calculated regional CBF values in seven small regions (including four subcortical structures and three cortical areas) of the brain are shown in Figure 7. The mean CBF values of SLIDER2/3/4 and reference across seven ROIs were 57.46 ± 6.89, 53.12 ± 7.57, 51.41 ± 6.74, and 56.51 ± 6.61 ml/100 g/min, respectively. Two-way ANOVA showed significant differences between these four conditions (*p* < 0.0001). The *post hoc* Wilcoxon signed-rank test showed no significant difference between SLIDER2 and reference (*p* = 0.59). There were significant differences between SLIDER3/4 and reference (*p* < 0.05 for SLIDER3 and *p* < 0.005 for SLIDER4.

### Spatial Blurring Evaluation

Table 2 shows the spatial blurring effect of SLIDER reconstructions, indicated by the correlation coefficient of the reconstructed image volume and the volume shifted one slice down in each subject. Two-way ANOVA showed that both SLIDER reconstructions and increased λ caused blurring in the slice direction, compared to the reference ASL images (*p* < 0.0001 for both factors). The *post hoc* Wilcoxon signed-rank test showed that SLIDER2 with the regularization parameter of λ = 0.1 had a minimum blurring effect with no statistical difference compared to the reference image (*p* = 0.88). SLIDER3 with λ = 0.1 slightly increased the slice correlation (*p* < 0.05), and SLIDER4 increased more (*p* < 0.01). The *post hoc* Wilcoxon signed-rank test also showed that the regularization parameter λ had influences on the slice blurring effects, with greater blurring effects induced by a larger regularization parameter (*p* < 0.01 was found between two λs in all SLIDER conditions).

**TABLE 2:** Slice blurring evaluation using autocorrelation of the reconstructed volume.

**Correlation**	**SLIDER2 λ = 0.1**	**SLIDER2 λ = 0.3**	**SLIDER2 λ = 0.5**	**SLIDER3 λ = 0.1**	**SLIDER3 λ = 0.3**	**SLIDER3 λ = 0.5**	**SLIDER4 λ = 0.1**	**SLIDER4 λ = 0.3**	**SLIDER4 λ = 0.5**	**Reference ASL**
Sub1	0.40	0.60	0.67	0.48	0.71	0.78	0.59	0.81	0.86	0.42
Sub2	0.41	0.55	0.62	0.51	0.72	0.78	0.63	0.82	0.87	0.41
Sub3	0.33	0.57	0.65	0.45	0.73	0.8	0.47	0.77	0.84	0.24
Sub4	0.43	0.64	0.7	0.55	0.78	0.84	0.64	0.85	0.9	0.40
Sub5	0.25	0.51	0.59	0.37	0.67	0.76	0.52	0.79	0.86	0.32
Mean ± SD	0.36 ± 0.07	0.57 ± 0.05	0.65 ± 0.04	0.47 ± 0.07	0.72 ± 0.04	0.80 ± 0.03	0.57 ± 0.07	0.81 ± 0.03	0.87 ± 0.02	0.36 ± 0.08

*λ is the regularization parameter used in SLIDER reconstruction.*

To further investigate the relationship between spatial blurring and SNR, additional slice blurring was applied using a Gaussian kernel with the full width at half maximum (FWHM) of 1.16 voxels for SLIDER2, 1.06 voxels for SLIDER3, and 1.18 voxels for the reference ASL image along the slice direction on SLIDER2/3 and reference ASL images to match that of SLIDER4 (see Supplementary Table 2 for burring evaluation before and after applying Gaussian kernel). Figure 5 shows the spatial and temporal SNR before and after applying the Gaussian kernel (the details of subjects are included in Table 1). As shown in Figure 5, SLIDER2/3/4 have similar sSNR after matching the level of spatial blurring, which still outperforms that of the reference ASL scan (*p* < 0.001). For tSNR, SLIDER4 still has the highest tSNR, while SLIDER3, SLIDER2, and reference scans have lower tSNR (*p* < 0.01).

## Discussion

### Advantages of Slice Dithered Enhanced Resolution-Simultaneous Multi-Slice Arterial Spin Labeling

In this work we presented super-resolution perfusion imaging using SLIDER and SMS pCASL with an optimized CSD-BS scheme. The SLIDER-reconstructed super-resolution images had significantly higher spatial and temporal SNR compared to the reference ASL images. The results are consistent with the theoretical estimation of SNR efficiency improvements due to super-resolution acquisitions of thicker slices. Since the number of slices that can be acquired for ASL is limited by TR and T1 relaxation, using a thicker slice will result in not only higher SNR but also a larger spatial coverage in the slice direction. This is shown in Figure 4, where SLIDER2/3/4 had a coverage of 96, 144, and 192 mm, respectively.

In this study, SLIDER reconstruction is found to improve the spatial and temporal SNR of reference ASL images, and the SNR improvement is greater with increased number of shifted slice groups. We also controlled the total scan time (∼14 min) to be identical across the three SLIDER methods and reference ASL for a fair comparison of SNR efficiency. The calculated g-factors were similar for SLIDER2, 3, and 4 with 20, 30, and 40 band spacing, respectively, suggesting a minor role the g-factor plays in determining the SNR of SLIDER ASL techniques in our study (see Eq. 4). While thermal random noise can be effectively suppressed by averaging following the rule of square root of N measurements, physiological noise is more complex with temporal correlations at specific frequencies. Therefore, noise reduction through averaging is less effective for physiological noise than thermal noise. These factors may have contributed to a higher SNR efficiency for SLIDER reconstructions with a high number of shifted slice groups. However, we did not systematically characterize thermal and physiological noise during ASL scans which should be performed in future studies (Moeller et al., 2006).

One advantage of the proposed SLIDER4 ASL technique is its wide coverage of 192 mm in the slice direction. There are some potential applications that may take advantage of such large coverage. For example, the labeling plane can be visualized with this method in coronal and sagittal images, which is not available for most of the ASL scans. In addition, B0 field inhomogeneity may distort the labeling plane; therefore, there is value to directly visualize the labeling plane. On the other hand, such large coverage would potentially allow measurement of perfusion in deep brain structures such as the brain stem, cerebellum, and even the spinal cord. These deep brain structures are key hubs of the structural and functional networks of the human brain, but so far very few perfusion measurements have been performed in these key deep brain structures.

### Spatial Blurring of Slice Dithered Enhanced Resolution-Simultaneous Multi-Slice Arterial Spin Labeling

The spatial blurring effect should be taken into consideration when evaluating the results. Table 2 shows that SLIDER reconstructions resulted in a small degree of slice blurring which increased with a greater number of shifted slice groups. A larger regularization parameter λ also caused a greater degree of slice blurring and reduced slice resolution as well as data fidelity. When additional slice blurring was applied, the improvement of sSNR of all the three SLIDER methods reached a similar level. For tSNR, however, SLIDER4 remained superior to SLIDER2/3 with matched slice blurring. This is because tSNR is prone to be affected by temporal fluctuation such as physiological noise while spatial blurring has more impact on sSNR. In a previous study on SLIDER, the authors suggested using λ∼0.1 which will not cause much blurring effect (Setsompop et al., 2015). Based on our experience, we also recommend the use of λ∼0.1 for SLIDER ASL as a tradeoff between imaging resolution and SNR.

Supplementary Figure 5 shows American College of Radiology (ACR) phantom images reconstructed with SLIDER2/3/4 (λ = 0.1) in comparison with reference phantom images. A small degree of spatial blurring can be observed in SLIDER3/4 reconstructed phantom images, which is consistent with our *in vivo* results on SLIDER ASL. From the quantitative CBF results in seven small brain structures, we found that the mean CBF values were reduced using SLIDER3/4 compared to the reference scan, likely due to blurring effects of SLIDER3/4 reconstructions. Although there was no significant difference between SLIDER2 and reference, the small sample size of our study may be a factor. Therefore, we recommend caution on quantifying and controlling blurring effects of SLIDER reconstruction for CBF quantification using the proposed SLIDER ASL method.

### Limitations of Slice Dithered Enhanced Resolution-Simultaneous Multi-Slice Arterial Spin Labeling

There are several limitations of our study. First, as a pilot study our sample size is small. There are some restrictions with the imaging protocol, e.g., there was no slice gap applied as the SLIDER reconstruction requires overlapping thick slices to resolve the thin slices. This may potentially induce slice cross talk and saturation effects. An alternative approach to solve this problem would be to improve the slice profile with longer RF duration or using Shinnar–Le-Roux (SLR) pulse design (Setsompop et al., 2018). We only acquired six MB slice groups within a TR in all of ASL scans, resulting in two passes for the reference high-resolution ASL scan. The main reason is that the efficiency of the CSD-BS scheme for suppressing background signals will decrease with more MB slice groups. Also, the total scan time of our method is around 14 min, making it susceptible to motion artifacts. This problem could be addressed with advanced imaging acceleration methods and image denoising methods such as deep learning (Xie et al., 2020). We also acknowledge that absolute CBF quantification using the proposed SLIDER ASL method awaits further validation since we observed variations in CBF across scan conditions in small brain structures. We also notice that MB factors higher than 4 can be used for larger number of slices and alternative methods for SNR calculation such bootstrapping (Riffe et al., 2007).

### Future Directions

In this work, we utilized SLIDER for the super-resolution reconstruction of ASL images to achieve isotropic-2-mm resolution perfusion images. Other more advanced super-resolution techniques such as gSLIDER (Setsompop et al., 2018) have been proposed for diffusion MRI. gSLIDER uses Hadamard encoding to achieve orthogonal basis; therefore, no regularization is needed for image reconstruction. For ASL, SLIDER SMS offers the technical simplicity and advantage for rapidly acquiring whole-brain images. The comparison of SLIDER and gSLIDER ASL may be included in future works. Further studies may also compare SLIDER reconstruction with high-resolution 3D ASL acquisitions, since 3D data tend to have higher SNR with larger FOV but with more slice blurring (FWHM on the order of 1.5 voxel size) (Vidorreta et al., 2014).

## Conclusion

We presented super-resolution perfusion imaging using SLIDER with 2D SMS pCASL. In conjunction with optimized CSD-BS, this technique can push the resolution of whole-brain ASL imaging to isotropic-2 mm and higher for fine-grained studies on brain perfusion. Caution needs to be exercised on quantifying and controlling blurring effects of SLIDER reconstruction.

## Data Availability Statement

The raw data supporting the conclusions of this article will be made available by the authors, without undue reservation.

## Ethics Statement

The studies involving human participants were reviewed and approved by the Institutional Review Board of the University of Southern California. The patients/participants provided their written informed consent to participate in this study.

## Author Contributions

QS designed the experiments and conducted data processing and analysis. XS helped with data processing and provided support for improvements on experiments. DW designed and supervised the experiments. All authors drafted the manuscript.

## Conflict of Interest

The authors declare that the research was conducted in the absence of any commercial or financial relationships that could be construed as a potential conflict of interest.

## Publisher’s Note

All claims expressed in this article are solely those of the authors and do not necessarily represent those of their affiliated organizations, or those of the publisher, the editors and the reviewers. Any product that may be evaluated in this article, or claim that may be made by its manufacturer, is not guaranteed or endorsed by the publisher.

## Footnotes

1
fil.ion.ucl.ac.uk/spm/software/spm12
