# Supplementary material for: Super-Resolution Arterial Spin Labeling Using Slice-Dithered Enhanced Resolution and Simultaneous Multi-Slice Acquisition
Source: Front Neurosci. 2021 Oct 29;15:737525. doi: 10.3389/fnins.2021.737525 (PMC8585855; doi:10.3389/fnins.2021.737525)
Supplement: Supplementary file 1 [file Data_Sheet_1.docx]

Supplementary Material

# Supplementary Figures and Tables

## Supplementary Figures


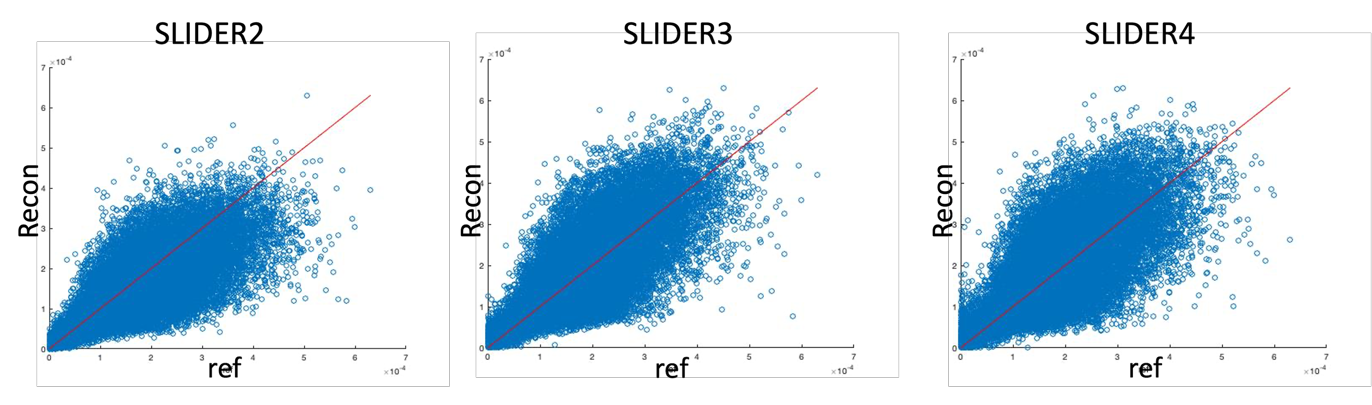


**Supplementary Figure S1.** Scatter plots of pixel-wise correlations between the SLIDER and reference ASL images within brain mask.


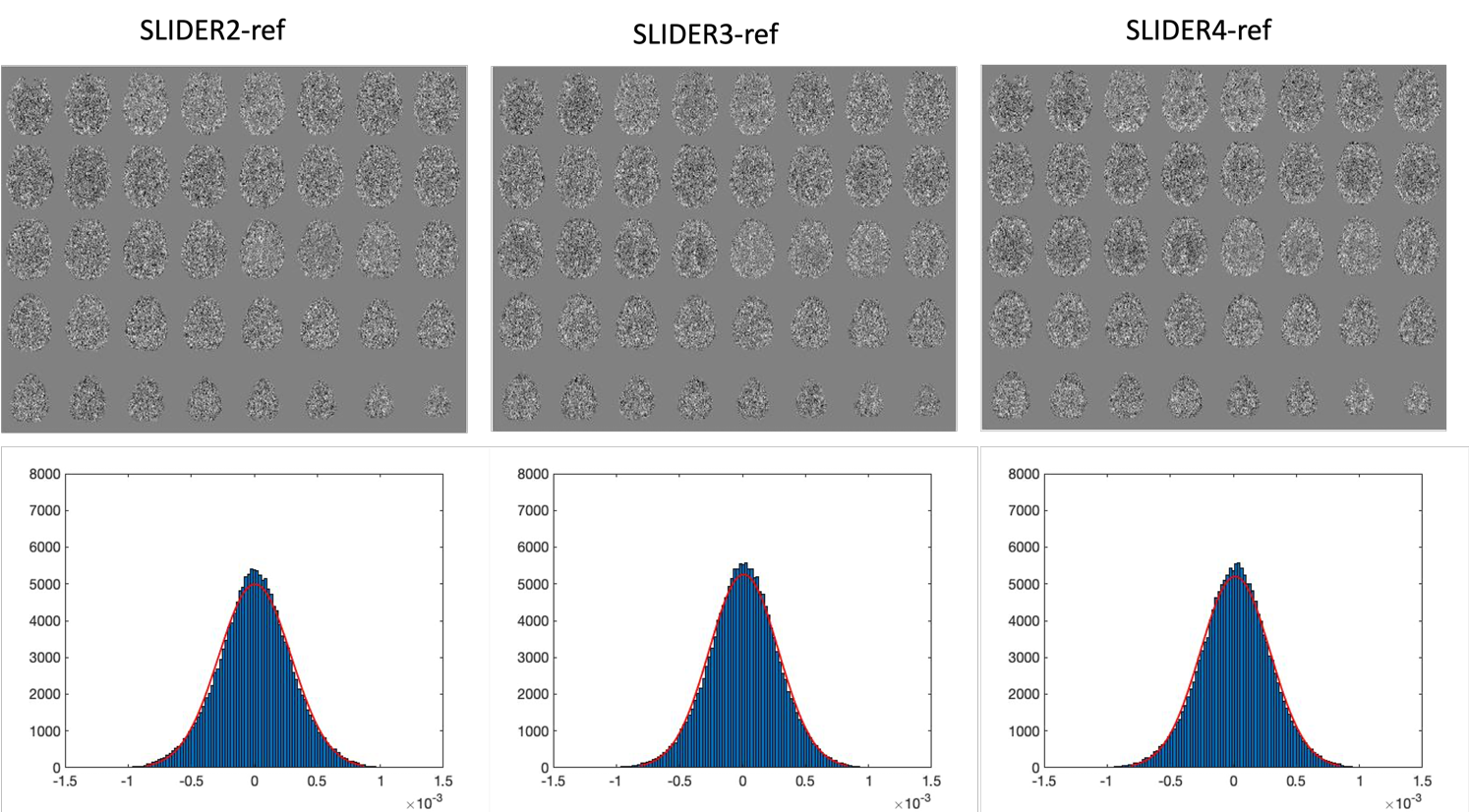


**Supplementary Figure S2.** Difference images between the SLIDER and reference ASL images along with the associated histograms of pixel values within brain mask.

**
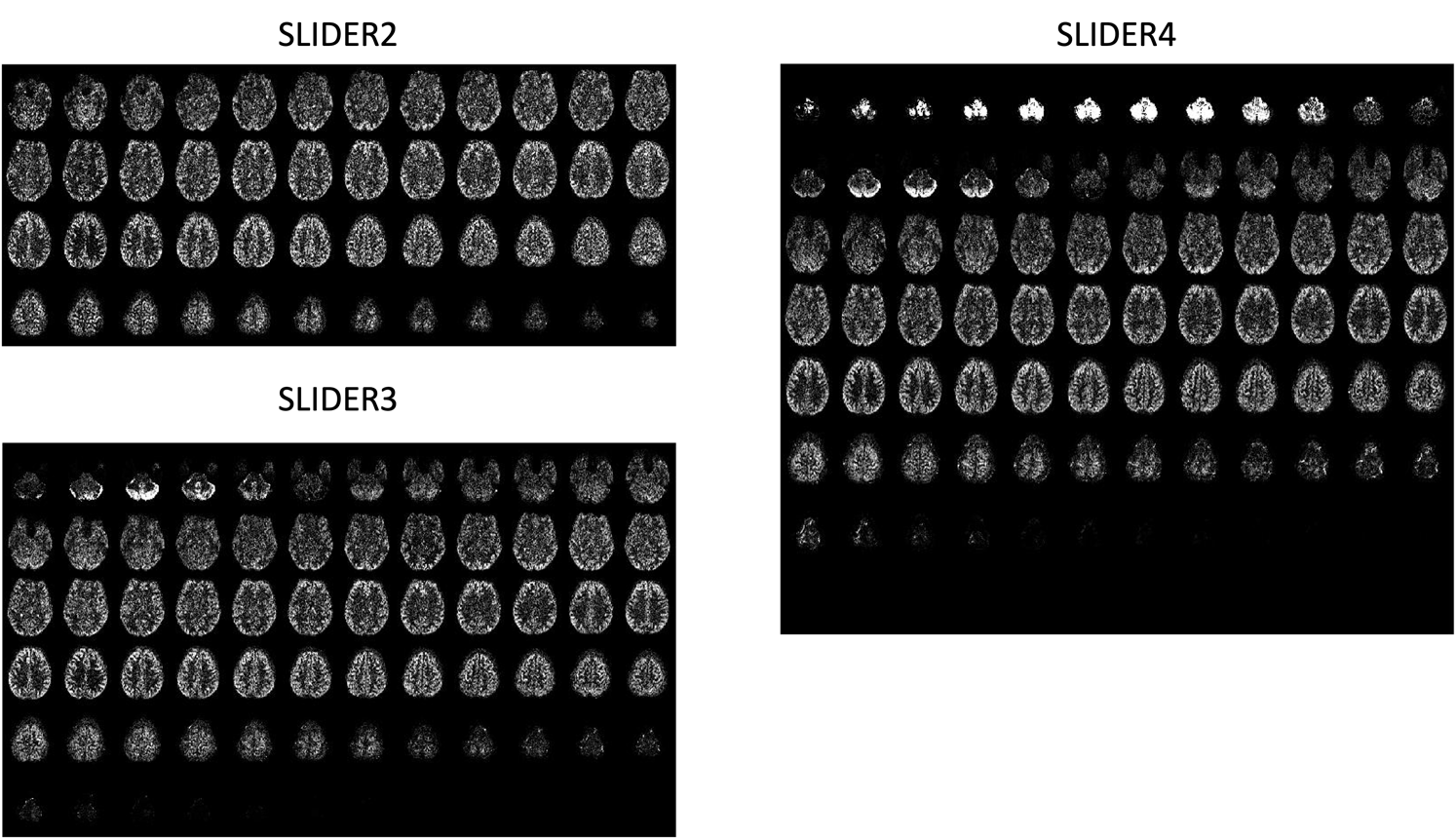
**

**Supplementary Figure S3.** All slices reconstructed in different SLIDER conditions. SLIDER2 has 48 slices, SLIDER3 has 72 slices and SLIDER4 has 96 slices.


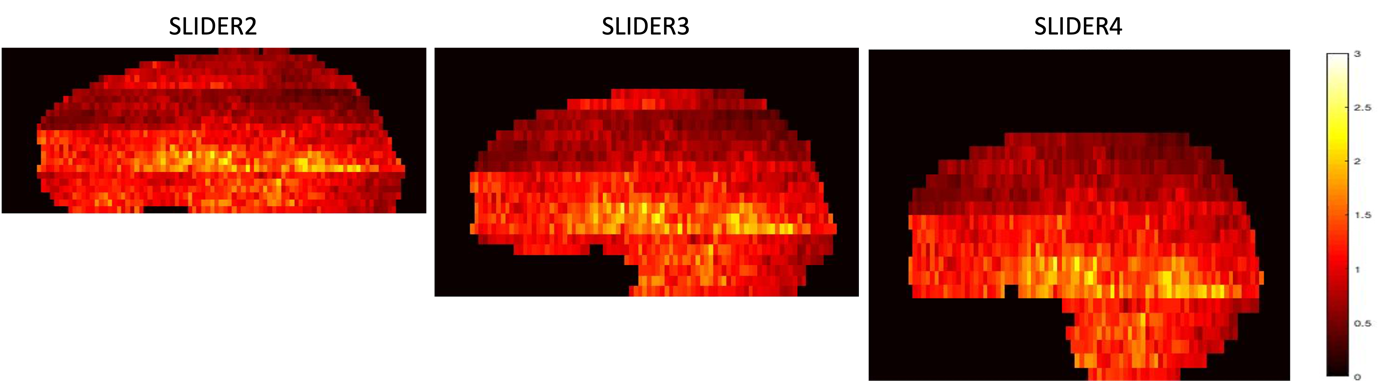


**Supplementary Figure S4.** G-factor maps for SLIDER2, SLIDER3 and SLIDER4 SMS acquisitions in the sagittal plane.

**
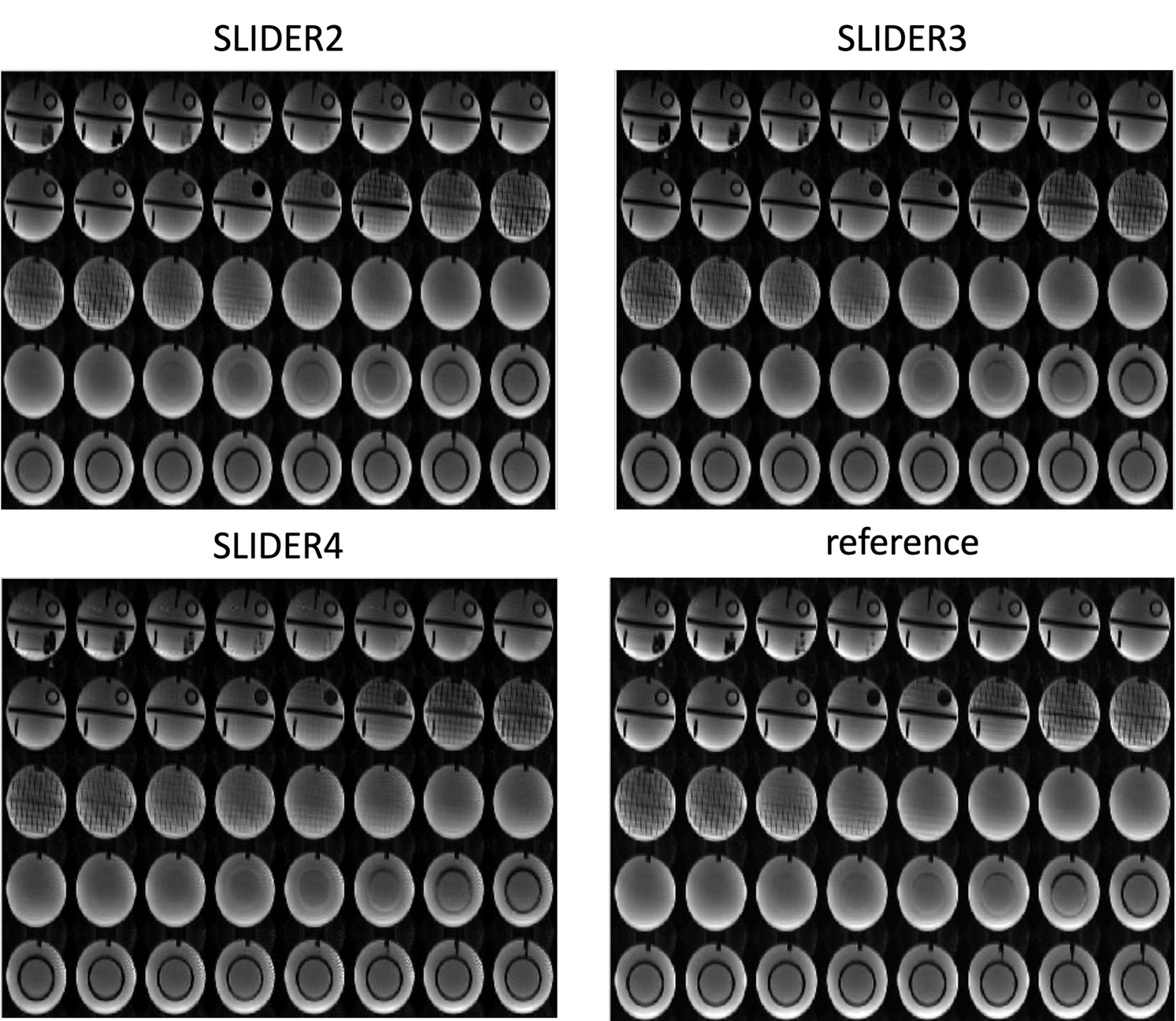
**

**Supplementary Figure S5.** SLIDER reconstructed images and reference images using a ACR phantom. Resolutions for all images are 2×2×2 mm^3^

**Supplementary Table S1.** Spatial and temporal SNR with and without applying PCA denoising method.

|  | sSNR PCA | sSNR  no PCA | tSNR  PCA | tSNR  no PCA |
| --- | --- | --- | --- | --- |
| sub1(SLIDER2) | 3.06 | 2.19 | 0.49 | 0.36 |
| sub1(SLIDER3) | 3.19 | 2.30 | 0.62 | 0.47 |
| sub1(SLIDER4) | 3.68 | 2.64 | 0.81 | 0.61 |
| sub1(reference) | 2.13 | 1.51 | 0.39 | 0.30 |
|  |  |  |  |  |
| sub2(SLIDER2) | 3.17 | 2.28 | 0.51 | 0.38 |
| sub2(SLIDER3) | 3.36 | 2.42 | 0.69 | 0.51 |
| sub2(SLIDER4) | 3.45 | 2.39 | 0.82 | 0.62 |
| sub2(reference) | 2.06 | 1.48 | 0.40 | 0.29 |
|  |  |  |  |  |
| sub3(SLIDER2) | 2.18 | 1.56 | 0.37 | 0.28 |
| sub3(SLIDER3) | 2.76 | 1.92 | 0.48 | 0.35 |
| sub3(SLIDER4) | 2.74 | 1.75 | 0.59 | 0.41 |
| sub3(reference) | 1.35 | 0.97 | 0.32 | 0.24 |
|  |  |  |  |  |
| sub4(SLIDER2) | 3.28 | 2.40 | 0.48 | 0.36 |
| sub4(SLIDER3) | 3.77 | 2.84 | 0.65 | 0.49 |
| sub4(SLIDER4) | 4.84 | 3.45 | 0.95 | 0.71 |
| sub4(reference) | 2.40 | 1.75 | 0.40 | 0.30 |
|  |  |  |  |  |
| sub5(SLIDER2) | 2.17 | 1.55 | 0.38 | 0.28 |
| sub5(SLIDER3) | 2.63 | 1.90 | 0.50 | 0.37 |
| sub5(SLIDER4) | 3.66 | 2.57 | 0.73 | 0.52 |
| sub5(reference) | 1.83 | 1.31 | 0.37 | 0.28 |
| mean± std (SLIDER2) | 2.77±0.55 | 2.00±0.41 | 0.45±0.07 | 0.33 ±0.05 |
| mean± std (SLIDER3) | 3.14±0.46 | 2.28±0.39 | 0.59±0.10 | 0.44±0.07 |
| mean± std (SLIDER4) | 3.67±0.76 | 2.56±0.61 | 0.78±0.13 | 0.57±0.11 |
| mean± std (reference) | 1.95±0.40 | 1.40±0.29 | 0.38±0.03 | 0.28±0.02 |

For the data without PCA denoising, similar improvements in sSNR and tSNR were observed. Kruskal-Wallis test showed significant difference between four conditions (P<0.01 for sSNR and P<0.005 for tSNR). Post-hoc Wilcoxon signed rank test showed significant higher sSNR for SLIDER2/3/4 and reference (P<0.05 for SLIDER2, P<0.01 for SLIDER3/4) and significant higher tSNR for SLIDER3/4 and reference (P<0.01). There is no significant difference in tSNR between SLIDER2 and reference.

**Supplementary Table S2.** Correlation before using Gaussian kernel and after using Gaussian kernel to control the blurring of each condition match SLIDER4.

| before control blur | sub1 | sub2 | sub3 | sub4 | sub5 | mean ± std |
| --- | --- | --- | --- | --- | --- | --- |
| SLIDER2 | 0.40 | 0.41 | 0.33 | 0.43 | 0.25 | 0.36±0.07 |
| SLIDER3 | 0.48 | 0.51 | 0.45 | 0.55 | 0.37 | 0.47±0.07 |
| SLIDER4 | 0.59 | 0.63 | 0.47 | 0.64 | 0.52 | 0.57±0.07 |
| reference | 0.42 | 0.41 | 0.24 | 0.40 | 0.32 | 0.36±0.08 |
| after control blur |  |  |  |  |  |  |
| SLIDER2 | 0.61 | 0.62 | 0.57 | 0.64 | 0.51 | 0.59±0.05 |
| SLIDER3 | 0.57 | 0.59 | 0.55 | 0.64 | 0.49 | 0.57±0.06 |
| SLIDER4 | 0.59 | 0.63 | 0.47 | 0.64 | 0.52 | 0.57±0.07 |
| reference | 0.64 | 0.64 | 0.52 | 0.64 | 0.57 | 0.60±0.06 |
